# Systematic review of observational studies of the impact of cardiovascular risk factors on preeclampsia in sub-saharan Africa

**DOI:** 10.1186/s12884-021-03566-2

**Published:** 2021-01-30

**Authors:** Oleg Iris Hounkpatin, Salimanou Ariyoh Amidou, Yessito Corine Houehanou, Philippe Lacroix, Pierre Marie Preux, Dismand Stephan Houinato, Holy Bezanahary

**Affiliations:** 1 INSERM, U1094, Tropical Neuroepidemiology, Limoges, France; 2 Univ. Limoges, U1094, Tropical Neuroepidemiology, Institute of Epidemiology and Tropical Neurology, GEIST, Limoges, France; 3 IRD, Associated Unit, Tropical Neuroepidemiology, Limoges, France; 4 School of Health Sciences, Laboratory of Chronic Diseases Epidemiology (LEMACEN), University Abomey-Calavi, Cotonou, Benin; 5 Department of Vascular Medicine–Vascular Surgery, CHU Limoges, Limoges, France; 6 Department of Internal Medicine, CHU Limoges, Limoges, France

**Keywords:** Risk factors, Preeclampsia, Systematic review, Sub-Saharan Africa

## Abstract

**Background:**

Maternal mortality is a public health issue, particularly in low- and middle-income countries (LMIC). Sub-Saharan Africa (SSA) is the region most affected worldwide by maternal mortality, and preeclampsia is one of the main causes. We performed a systematic review of observational studies to identify the impact of cardiovascular risk factors on preeclampsia in SSA with a more representative sample.

**Methods:**

Databases: PubMed and Google Scholar were searched to identify published studies. Studies were included if they reported results on the link between at least one cardiovascular risk factor and preeclampsia. Relevant studies quality was assessed with the Newcastle-Ottawa Scale (NOS). Odds ratios and relative risk (RR) were reported with their confidence intervals.

**Results:**

Twelve articles (8 case-controls, 3 cohorts, 1 cross-sectional) were included in this review, with a total of 24,369 pregnant women. Cardiovascular risk factors such as chronic hypertension, overweight, obesity, diabetes and alcohol were significantly associated with a high risk of preeclampsia. Very few data were available concerning some risk factors. None of the articles reported tobacco consumption as a preeclampsia risk factor. There is a lack of data from French-speaking SSA countries.

**Conclusion:**

Cardiovascular risk factors increase the risk of preeclampsia. Our results suggest the need for prospective cohort studies to ascertain this association in order to reduce maternal mortality due to preeclampsia.

## Background

Maternal mortality is a public health issue, particularly in low- and middle-income countries (LMIC). Although the number of maternal deaths worldwide has decreased by 45% since 1990, 800 women still die every day from largely preventable causes before, during and after childbirth, with disparities by region [1]. Sub-Saharan Africa (SSA) is the most affected region, with 66% of maternal deaths. Hypertensive disorders during pregnancy were identified as the second leading cause, after hemorrhage, of maternal and perinatal death, accounting for 14 and 27.1%, respectively [2]. According to the World Health Organization (WHO), 16% of maternal deaths in sub-Saharan Africa are attributable to hypertensive disorders during pregnancy, with preeclampsia and eclampsia being the leading causes [3]. The incidence of preeclampsia ranges from 3 to 5% [4] and can reach 10% [5], depending on the region, and remains higher in LMIC. Preeclampsia increases maternal and perinatal mortality through both fetal and maternal complications. In the literature, authors reported the involvement of many risk factors; the most frequently cited are advanced age, multiple pregnancies, nulliparity, personal history of preeclampsia and pre-pregnancy medical conditions such as chronic high blood pressure, type 2 diabetes and renal failure [6, 7]. In the last years, with the spread of cardiovascular diseases, many studies have reported that cardiovascular risk factors are involved in the onset of preeclampsia [8–11]. However, most of these studies were conducted in developed countries. In SSA, a large majority were conducted in English-speaking countries. To estimate the real contribution of cardiovascular risk factors, it is necessary to provide representative data. Moreover, especially about SSA countries, that have one of the highest maternal mortality rates worldwide. Since most of these risk factors are preventable, early identification of women at risk appears to be mandatory for optimal management.

This study aimed to systematically review all observational evidence regarding the impact of cardiovascular risk factors on preeclampsia in SSA.

## Methods

### Search strategy

This review was conducted according to PRISMA guidelines [12]. The study protocol was approved by the review team before searching was performed. Studies were retrieved through internet research in PubMed and Google Scholar databases from March 6th to April 30th, 2019**.** Searching was performed by using terms in line with the “PICO” method**.** The search was done with the following terms: hypertension, diabetes mellitus, overweight, obesity, tobacco use, alcohol consumption, dyslipidemia, preeclampsia, hypertensive disorders, Africa South of Sahara, and the names of all SSA countries. Keywords were used as follow: ((“hypertension” OR “diabetes mellitus” OR “overweight” OR “obesity” OR “alcohol consumption” OR “tobacco use” OR “dyslipidemia”) AND (preeclampsia OR “hypertensive disorders”)) and all SSA countries names. References of relevant studies were manually screened for additional articles. Titles and abstracts were screened first. Then a full quality assessment review of articles was done before selection for final review. Two authors independently assessed the articles for inclusion in the review. Any discrepancy which arose between the authors in the review process was resolved through discussion for consensus.

### Eligibility criteria

Studies were included in the review if:

- the design was cross-sectional, case-control, prospective or retrospective cohorts,
- it evaluated the link between at least one cardiovascular risk factor (chronic hypertension, diabetes, dyslipidemia, obesity, overweight, tobacco, alcohol) and preeclampsia.
- it was published in English or French.

We did not use a restriction on the years of publication. Only studies meeting our inclusion criteria were selected. The quality of retrieved studies was assessed using the Newcastle-Ottawa scale (NOS) [13]. Selection, comparability, and outcome/exposure assessment were rated for case-control and cohort studies separately. The rating system scores from 0 (highest degree of bias) to 9 (lowest degree of bias) allocated among three criteria: selection (0 to 4), comparability (0 to 2) and outcome/exposure assessment (0 to 3). Studies with a total score of 0 for one of the three criteria were excluded.

### Data extraction

Selected studies were analyzed for data extraction. We extracted data concerning: authors, years and areas of studies, study populations, study design, sample size, sampling methods, inclusion and exclusion criteria, preeclampsia definition, risk factors, length of follow up for cohort studies and exposure variables for all studies. We also reported all studied variables per each article. Targeted risk factors were chronic hypertension, diabetes mellitus, overweight, obesity, alcohol, tobacco, and dyslipidemia. Preeclampsia was a main outcome reported in the included studies. Bias, confounding and their management were assessed in included studies by reviewing each study methods and discussion sections. Studies with significant bias were not included in this review. When available, results were reported as odds ratios or relative risks with 95% confidence intervals.

### Outcomes

The main outcome was preeclampsia. It was defined as new onset of systolic blood pressure of 140 mmHg or more and diastolic blood pressure of 90 mmHg or more, at least twice on two occasions at least 4 h apart, with either 24 h proteinuria ≥300 mg or urinary protein strip showing ≥1+ without urinary tract infection, according to the American College of Obstetricians and Gynecologists, in almost all the included studies. However, one study used a higher blood pressure cut-off of 160/110 associated with random proteinuria ≥2+ [14].

## Results

**Fig. 1:**
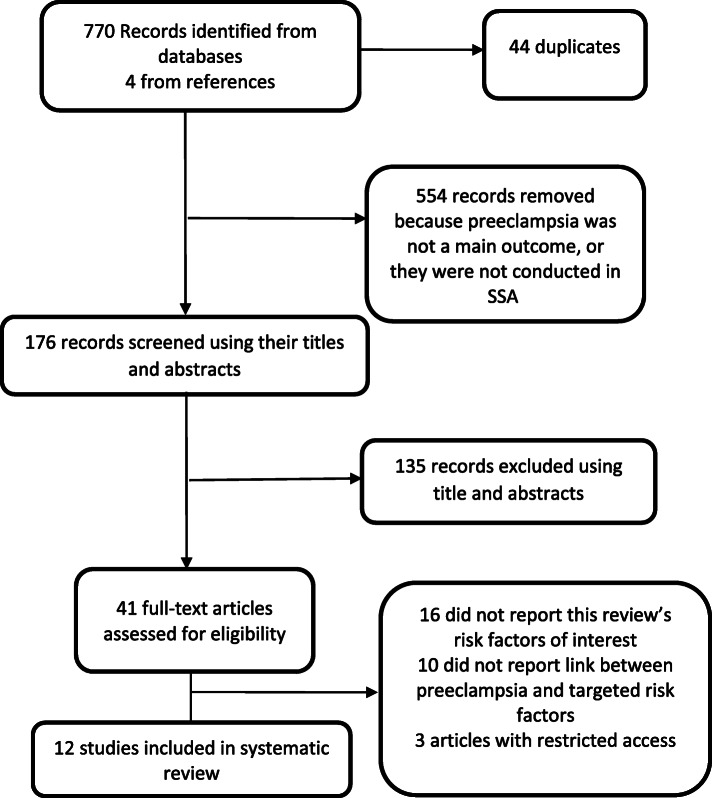
Flow diagram for selection of studies included in systematic review

A total of 770 articles were retrieved through electronic searching in databases and 4 studies from references. Of this, 44 duplicates were removed and 554 studies were removed because they were conducted in a region other than SSA, or preeclampsia (hypertensive disorders) was not a main outcome. One hundred and seventy-six titles and abstracts were screened. Finally, twelve studies (8 case-control, 3 cohorts and one cross-sectional) were included in this systematic review. The study selection process is summarized in Fig. 1.

Most studies had high quality case definition and subject selection. Cases and controls were selected from the same population. There was no information on the proportion of non-response and the representativeness of the sample in some studies. All included studies scored 7 and above at quality assessment. Table 1 and Table 2 present NOS-based evaluation of case-control and cohort studies, respectively.

**Table 1:** Quality assessment of included case-control studies

	Mahomed et al.	Anorlu et al.	Kiondo et al.	Guerrier et al.	Endeshaw et al.	Grum et al.	Kahsay et al.	Mrema et al.
Selection								
Is the case definition adequate?	1	1	1	1	1	1	1	1
Representativeness of cases	1	1	1	1	1	1	0	1
Selection of Controls	0	0	0	0	0	0	0	0
Definition of Controls	1	1	1	1	1	1	1	1
Comparability								
Comparability of cases and controls based on the design or analysis	2	2	1	2	2	2	2	2
Exposure								
Ascertainment of exposure	1	1	1	1	1	1	1	1
Same method of ascertainment for cases and controls	1	1	1	1	1	1	1	1
Non-Response rate	0	1	1	0	1	1	1	0
Total	7	8	7	7	8	8	7	7

**Table 2:** Quality assessment of included cohort studies

	Singh et al.	Baragou et al.	Musa et al.
Selection			
Representativeness of the exposed cohort	1	1	1
Selection of the non-exposed cohort	0	1	1
Ascertainment of exposure	1	1	1
Demonstration that outcome of interest was not present at start of study	1	1	1
Comparability			
Comparability of cohorts based on the design or analysis	2	2	2
Outcome			
Assessment of outcome	1	1	1
Was follow-up long enough for outcomes to occur?	1	1	1
Adequacy of follow-up of cohorts	1	1	1
Total	8	9	9

### Studies description

The characteristics of included studies are shown in Table 3. They were conducted between 1998 and 2018. Participants were recruited during pregnancy or after delivery. Preeclampsia standards were not identical among all included studies. They were based either on the American College of Obstetrician and Gynecologists or the International Society for the Study of Hypertension in Pregnancy (ISSHP) standards. Only one study was conducted in a French-speaking SSA country. In cohort studies, pregnant women were included before 20 weeks of pregnancy. Case-control study populations mostly included women who were more than 20 weeks pregnant or those who had already given birth. Some cardiovascular risk factors such as dyslipidemia and tobacco use were not studied. Few studies assessed tobacco (4/12). Sample size was calculated in almost all the included studies. The odds ratios are summarized according to the risk factors in Table 4.

**Table 3:** Characteristics and risk factors reported in included studies

References	Setting	N	Cases	Study Population	PE assessment	Predictor variables	Risk factors
**Case controls**							
Mohamed et al.1998 [15]	Harare, Zimbabwe	338	144 cases, 194 controls	Women who delivered at Harare hospital maternity from June 1995 to April 1996, recruited from admission and birth records. Controls were normotensive pregnant women delivering within two hours and were matched in childbirth mode with the case.	American College of Obstetricians and Gynecologists definition Blood pressure > 140/90 mmHg at least twice and occasional proteinuria > 1 g/l at least twice at 4 h intervals.	Age, overweight, obesity.	Overweight OR **7** (2.9–19.4)
Anorlu et al. 2005 [6]	Lagos, Nigeria	368	128 cases, 240 controls	Delivered women at Lagos university hospital from February 2001 to August 2002 with preeclampsia or eclampsia. The randomly selected controls were women who gave birth at 24–48 h intervals without preeclampsia. For each case, two controls were selected.	Blood pressure > 140/90 mmHg at least twice on two occasions at least 4 h apart with either 24 h proteinuria ≥300 mg or urinary protein strip with ≥1+ protein in the absence of urinary tract infections.	Age, parity, educational level, occupation, home and work environment, family history of hypertension, chronic hypertension, previous preeclampsia.	Chronic hypertension OR **2.21** (1.17–6.20)
Kiondo et al. 2012 [16]	Mulago, Uganda	557	207 cases, 352 controls	Pregnant women aged 15 to 39 randomly selected from computer database from May 1, 2008 to May 1, 2009 living within 15 km of Mulago hospital and more than 20 weeks pregnant. Women with cardiac disease, sickle cell disease, eclampsia, HELLP syndrome and renal failure were excluded from controls. For each identified case, two controls were selected on the same day until the required number of patients was obtained.	Blood pressure > 140/90 mmHg at least twice on two occasions at least 4 h apart with either 24 h proteinuria ≥300 mg or random proteinuria ≥1+	Age, educational level, marital status, smoking status, alcohol intake, MUAC, history of diabetes, hypertension, family history of hypertension, parity.	Chronic hypertension OR **2.29** CI (1.12–4.66)
Guerrier et al. 2013 [14]	Jahun, Nigeria	1676	419 cases (175 severe PE, 244 eclampsia), 1257 controls	Pregnant women admitted from October 2010 to May 2011.	Severe PE: Blood pressure > 160/110 mmHg and random proteinuria ≥2+	Age, ethnicity, occupation, personal history of PE, history of hypertension, family history of PE and hypertension, number of ANC visits.	Chronic hypertension OR **10.5** (5.8–19)
Endeshaw et al. 2016 [17]	Bahir Dar, Ethiopia	453	151 cases, 302 controls	Pregnant women attending or delivered in a referral hospital or six health centers in Bahir Dar between June and September 1994, diagnosed pre-eclamptic by a physician or midwife. For each case, two controls were randomly selected in the same center on the same day.	Blood pressure > 140/90 mmHg at least twice on two occasions and laboratory-confirmed proteinuria	Age, area of residence, marital status, occupation, MUAC, fruit, vegetable, folate, alcohol and coffee intake, anemia, physical activity.	Obesity OR **3.33** (1.87–5.79)
Grum et al. 2017 [18]	Addis Ababa, Ethiopia	291	97 cases, 194 controls	Pregnant women attending the two largest hospitals of Addis Ababa from December 2015 to February 2016 diagnosed pre-eclamptic by an obstetrician. Women with known hypertension, renal disease, with serious medical conditions and those who could not give consent were excluded. Controls were women not diagnosed with preeclampsia in the two hospitals.	Blood pressure > 140/90 mmHg at least twice on two occasions with either 24 h proteinuria ≥300 mg or random proteinuria ≥1+ after 28 weeks gestation	Age, ethnicity, religion, marital status, occupation, educational status, family history of hypertension, history of eclampsia, gravidity, multiple pregnancy, alcohol, fruit intake.	Alcohol consumption OR **3.97** (1.8–8.75)
Kahsay et al. 2018 [19]	Tigray, Ethiopia	330	110 cases, 220 controls	Pregnant women with gestational hypertension or PE diagnosed by an obstetrician in one of the seven hospitals in the Tigray region from June to November 2017. Women without hypertension were controls, women less than 20 weeks pregnant were excluded. Cases and controls were matched for parity and time of arrival at hospital.	Blood pressure ≥ 140/90 mmHg at least twice associated with proteinuria ≥1+ on the urine test strip	Age, area of residence, marital status, occupation, ethnicity, income category, family history of hypertension, MUAC, BMI, fruit and vegetable intake, coffee use, history of smoking, diabetes mellitus, oral contraceptive use.	Overweight OR **5.5** (1.12–27.6 Diabetes OR **5.4** (1.1–27.0)
Mrema et al. 2018 [10]	Kilimanjaro, Tanzania	17,738	582 cases, 17,156 controls	Birth records selected women with one previous pregnancy and a monofetal actual pregnancy diagnosed as pre-eclamptic by an obstetrician. Normotensives pregnant women were controls.	Blood pressure > 140/90 mmHg associated with 24 h proteinuria ≥300 mg	Age, marital status, occupation, educational level, pregnancy number, number of ANC, chronic hypertension, diabetes, heart disease, BMI.	Overweight OR **1.4** (1.2–1.8) Obesity OR **1.8** (1.3–2.4)
**Cohort studies**							
Singh et al. 2014 [20]	Sokoto, Nigeria	216	13 PE (6%)	Pregnant women attended Usmanu Danfodiyo University hospital from March to December 2011 at less than 20 weeks pregnancy and were followed-up to 6 weeks after giving birth. The main outcome was the development of hypertensive disorders during the follow-up period.	Blood pressure > 140/80 mmHg associated with proteinuria	Age, marital status, education, occupation, type of gestation, family history of hypertension and diabetes mellitus, history of preeclampsia, BMI.	Obesity RR **2.7** (1.3–5.7)
Baragou et al. 2014 [21]	Lomé, Togo Urban	1620	114 PE (7%)	Pregnant women recruited in the gynecology service of Tokoin University hospital from October 1, 2011 to September 31, 2012. The main outcome was hypertensive disorder during pregnancy. Women were followed during the pregnancy to 3 months postpartum.	Blood pressure > 140/90 mmHg at least twice on two occasions at least 4 h apart with either 24 h proteinuria ≥300 mg or urinary protein strip with ≥1+ protein in the absence of urinary tract infection	Age, occupation, multiple pregnancy, stress, obesity, familial history of hypertension, history of preeclampsia.	Chronic hypertension RR **3** Obesity RR **2.8**
Musa et al. 2018 [22]	Jos, Nigeria	307	27 PE (8.8%)	Pregnant women attending the Jos University hospital between November 2010 to August 2011 before 20 weeks pregnancy without any signs of PE. Patients with chronic hypertension, proteinuria, chronic renal failure, diabetes, sickle cell disease were not included. The primary outcome was preeclampsia and follow-up was terminated at any gestational age if the woman developed preeclampsia or delivery of her baby with or without development of preeclampsia.	Blood pressure ≥ 140/90 mmHg at least twice associated with proteinuria ≥2+ on the urine test strip	Age, gestational age, BMI, previous PE, miscarriages, parity, history of infertility, HIV status.	Obesity RR **3.9** (1.5–10.0)
Tessama et al. 2013 [23]	Dessie, Ethiopia	475	41 PE (8.6%)	Pregnant women attending prenatal visits in Dessie Hospital between August and September 2013 at more than 20 weeks of pregnancy.	Blood pressure > 140/90 mmHg associated with proteinuria ≥1+ on the urine test strip	Age, marital status, ethnicity, educational level, tobacco, alcohol use, family history of hypertension and diabetes mellitus, history of hypertension, gravidity, parity.	Chronic hypertension OR **4.3** (1,33-13,9)

*PE* Preeclampsia, *OR* Odds Ratio, *RR* Risk Ratio

**Table 4:** Reported Odds Ratios according to each risk factor

Risk factors	Number of studies	Odds Ratios/Relative Risk	95% Confidence Intervals
Chronic hypertension	5	**2.2**	1.17–6.20
		**2.3**	1.12–4.66
		**10.5**	5.8–19
		**4.3**	1.33–13.9
		**3.0**	
Obesity	5	**3.3**	1.87–5.79
		**1.8**	1.3–2.4
		**2.7**	1.3–5.7
		**2.8**	
		**3.9**	1.5–10.0
Overweight	3	**7.0**	2.9–19.4
		**5.5**	1.12–27.6
		**1.4**	1.2–1.8
Diabetes	1	**5.4**	1.1–27.0
Alcohol	1	**4.0**	1.8–8.75

### Chronic hypertension

Chronic hypertension increased the risk of developing preeclampsia by up to 10-fold [6, 14, 16, 21]. Some included studies did not evaluate for chronic hypertension [15, 18, 19].

### Diabetes

In our review, only one case-control study reported the role of pregestational diabetes in the occurrence of preeclampsia. Pregnant women with pre-gestational diabetes were four times at risk of developing preeclampsia [19]. Pregnant women with known diabetes before pregnancy were not included in some studies [22].

### Overweight and obesity

Three case-control studies reported that being overweight before pregnancy increased the risk of developing preeclampsia by up to seven times [10, 15, 22]. All cohort studies [20–22] and two case-control studies [10, 17] in this review reported that obesity was an important risk factor for preeclampsia.

### Alcohol

One case-control study suggested an association between alcohol and preeclampsia. In this study, women who reported alcohol consumption during pregnancy were more likely to develop preeclampsia compared to those who did not drink alcohol in multivariable analysis (aOR = 3.97, 95% CI = 1.80–8.75) [18]. However, Kiondo et al. found no association between alcohol and preeclampsia [16].

## Discussion

The prevalence of cardiovascular risk factors in SSA women is not negligeable. The median prevalences were: hypertension (29%), diabetes (7%), overweight (35%), obesity (11%), alcohol consumption (13%), tobacco (2%) [24–26]. Apart from tobacco, there is a significant prevalence of cardiovascular risk factors in SSA women.

This review presents evidence from published observational studies on the association between cardiovascular risk factors and preeclampsia. Chronic hypertension can increase the risk of developing preeclampsia during pregnancy by 3 to 10-fold. This association was also found among Latin American and Caribbean women and is generally present in LMIC [8, 27]. A possible explanation for this is the lack of follow-up of chronic hypertension among women. These results emphasize the need for detecting and managing hypertension among women in SSA.

Other high-risk factors for preeclampsia are overweight and obesity. These findings corroborate Proorolajal’s et al. meta-analysis, which concluded that overweight and obesity are predictors of preeclampsia. Paré et al., in a prospective cohort study, reported that the risk of preeclampsia increases with the body mass index [7, 28]. These results require clarification, as the BMI in the pregestational period could not be evaluated in most cases. In LMIC, women are not always able to accurately report their body mass index or weight, so some authors have used mid-upper arm circumference to assess nutritional status, because this parameter may be stable during pregnancy [29]. This methodological particularity could lead to an overestimation of obesity cases, because pregnancy is responsible for weight gain in most women. Other authors have evaluated BMI based on the weight reported by pregnant women, which is an important methodological limitation. Obesity is associated with oxidative stress, increased levels of circulating inflammation markers, dyslipidemia, insulin resistance and impaired endothelial function. These metabolic and biochemical disturbances likely predispose to an intrauterine environment favorable to placental perfusion disorders and endothelial dysfunction in preeclampsia [30].

Although only one study reported a significant association between pre-gestational diabetes and preeclampsia, our findings are similar to those reported by Duckitt et al. in a systematic review. Being diabetic before pregnancy increases the risk of preeclampsia by 4 times [31]. However, one study alone does not permit generalizing these findings. Like diabetes, alcohol consumption and dyslipidemia have been poorly studied. Both factors may increase the risk of preeclampsia [18]. However, Kiondo et al. found no association between alcohol consumption and preeclampsia in Uganda [16]. These results may be explained by the fact that alcohol consumption was self-reported, and in SSA and worldwide, alcohol consumption by pregnant women is not considered acceptable, or is even prohibited. No studies evaluated the impact of tobacco on the occurrence of preeclampsia. This may be explained by the fact that in SSA, tobacco use is not common among women. However, tobacco in its smoking form reduces the risk of preeclampsia by 35%, although the mechanism remains controversial [32]. This protective effect of smoking on preeclampsia risk is limited to low-risk pregnancies only. Smoking is an independent risk factor for superimposed preeclampsia in chronic hypertension, which is significant in the context of other cardiovascular risk factors [33]. Despite this lack of information, the harmful effects of tobacco on health, especially in arteriosclerosis, should not be neglected.

In LMIC, there is an epidemiological transition from communicable disease to non-communicable disease, led by cardiovascular disease, which accounted for 33.2% of deaths worldwide in 2008 [26]. Since cardiovascular risk factors are involved in the occurrence of preeclampsia, the screening of women at risk could help reduce mortality related to hypertensive complications of pregnancy, in particular preeclampsia. Reducing preeclampsia and cardiovascular risk factors would lessen long-term cardiovascular complications and therefore female mortality linked to non-communicable disease. Indeed, the occurrence of preeclampsia can increase by four the risk of heart failure and by two the risk of stroke, coronary heart disease or death from a cardiovascular event in the medium and long term.

However, this review has some limitations. The studies were all conducted in hospital settings. Therefore, there might be a selection bias because of home deliveries. The lack of studies in French speaking Sub-Saharan African countries does not permit generalizing our results. The self-reported data collected in the studies does not guarantee the reliability of the results. Some authors did not consider possible confounding factors that could lead to misestimation of their results. We included studies from various geographical areas, conducted with specific methodologies. One limitation of our review is that we report results from studies with potential limitations and which did not focus exclusively on preeclampsia.

## Conclusion

Cardiovascular risk factors are involved in the occurrence of preeclampsia in SSA. This review highlights the role of chronic hypertension, diabetes, overweight, obesity, alcohol. Some of the included studies have methodological limitations. The lack of data suggests a need for prospective cohort studies to assess the impact of cardiovascular risk factors on the occurrence of preeclampsia in SSA. Pre-pregnancy cardiovascular risk factor screening is needed to prevent preeclampsia among women.

## Data Availability

The datasets used and analyzed during the current study are available from the corresponding author on reasonable request.

## Abbreviations

SSA: Sub-Saharan Africa
LMIC: Low- and middle-income countries
NOS: Newcastle-Ottawa scale
WHO: World health Organization
PRISMA: Preferred reporting items for systematic reviews and meta-analyses
ISSHP: International society for the study of hypertension in pregnancy
BMI: Body mass index
